# Condylar response to large mandibular advancement combined with maxillary impaction and counterclockwise rotation: A computed tomographic study

**DOI:** 10.4317/jced.54933

**Published:** 2018-09-01

**Authors:** Francisco Vale, Inês Francisco, Jessica Scherzberg, Adriana Guimarães, Francisco Caramelo, Luísa Maló

**Affiliations:** 1 DMD, MSc, PhD. Professor and Chairman - Institute of Orthodontics, Faculty of Medicine - University of Coimbra; 2 DMD, MSc. Postgraduate student - Institute of Orthodontics, Faculty of Medicine - University of Coimbra; 3 MSc, PhD. Professor - Laboratory of Biostatistics and Medical Informatics, Faculty of Medicine - University of Coimbra; 4 DMD, MSc, PhD. Professor - Institute of Orthodontics, Faculty of Medicine - University of Coimbra

## Abstract

**Background:**

This study aims to analyze the effectiveness of cone-beam computed tomography (CBCT) in the evaluation of the condylar position, angulation and intercondylar distance and assess the changes in these parameters before and after bimaxillary surgery, preformed with the critical movments of Le Fort I osteotomy (for impaction of the maxilla and conterclockwise rotation of the upper occlusal plane) and Bilateral Sagittal Split Osteotomy (BSSO) for mandibular advancement (> 8mm).

**Material and Methods:**

Twenty class II patients successfully treated with BSSO of the mandible, in conjunction with Le Fort I osteotomy, were studied to evaluate the condylar changes before and after surgery. The position of the condyle was classified according to the Pullinger & Hollender’s formula in both phases. A MANOVA analysis followed by post-hoc tests were conducted to ascertain if there were statistically significant differences between pre and post surgical variables under study. The agreement of the condylar position’s classification was evaluated resorting to the Kappa statistics.

**Results:**

There were no statistically significant differences between the values of the position and angulation of the condyles and intercondylar distance before and after surgery. There was an increase of the axial angle of the left condyle and the frontal angle of both condyles, while there was a decrease of the axial angle of the right condyle, the sagittal angle of both condyles and intercondylar distance.

**Conclusions:**

The CBCT is a useful method for assessing variations of condylar position in detail. It was verified that the critical movements of maxillary impaction associated with the mandibular advancement do not produce significant alterations in the mandibular condyles, however, these tend to perform a posterior and inferior movement.

** Key words:**Cone-Beam computed tomography, orthognathic surgery, mandibular condyle, osteotomy, le fort, temporomandibular joint.

## Introduction

The optimal position of the condyle in the glenoid fossa, when the teeth are in centric relation, is a key factor in the functional stability of the stomatognathic system and has been widely discussed (1). The functional forces applied to the temporomandibular joint (TMJ) may affect the morphology therefore a relation between function and structure exists. Because these forces vary with the dento-facial morphology of the individual, the condyle and the glenoid fossa may have different shapes depending on the type of occlusion present (2).

Conventional radiographs of the TMJ have limitations regarding the accurate assessment of the condylar position. This is due to the fact that it is a small joint with a complex morphology surrounded by bone tissue, leading to overlay images, particularly in the petrous region of the temporal bone, mastoid process and articular eminence (3,4).

In orthodontics, CBCT may have several practical applications. Regarding the evaluation of the TMJ, the CBCT images reproduce the temporomandibular joint with great accuracy and are more reliable and accurate in the detection of condylar resorption than CT or panoramic views (3,5).

The objectives of combined orthodontic-surgical treatment of dentofacial deformities are to improve facial aesthetics, maintaining a static and functionally healthy occlusion and stable results (6). A good occlusal relationship and a normal condylar position after orthognathic surgery are considered important factors in preventing a postoperative relapse. Although a stable occlusion can be maintained in the pre and post-surgical orthodontic treatment, the condylar position is a difficult variable to control during and after surgery (3,6,7).

The factors causing condyle position changes during orthognathic surgery include the patient’s posture during surgery, muscle relaxation through the use of muscle relaxants, an inadequate rigid fixation, edema or intracapsular bleeding, asymmetrical surgical movement, or a combination of these factors (8). Also the positioning of the condyle after surgery can be affected by several factors such as the direction and amount of movement of the distal segment, the anatomical shape and orientation of the proximal segment, the tensional balance of the adjacent muscles, the fixation method applied and the surgeon`s experience (9).

Regarding the stability after orthognathic surgery, two different situations can occur: an early relapse, which takes place in the first months after surgery, or a late relapse (10,11). The relapse can occur in the osteotomy site, due to inter-segment movements, and in the TMJ, due to condylar distraction, rotation of the proximal segment or morphological changes in the condyle (12).

Although most of the surgical techniques are considered to be highly stable, especially in single-jaw procedures, other complications may arise in addition to postoperative relapse (13-15). An inappropriate condylar position can also lead to idiopathic condylar resorption, appearance or worsening of temporomandibular disorders (TMD) and anterior open bites. In addition to its stability, maintenance of the condyles in the correct position is essential for preventing dissatisfaction with surgical outcomes (1,3,8,13,15-17).

Condylar resorption after orthognathic surgery may occur after all types of osteotomy: after bimaxillary surgery in 68% of the cases, after BSSO in 25% of the cases and after isolated Le Fort I osteotomy in 7% of the cases. Impaction of the maxilla and counterclockwise rotation of the upper occlusal plane are at-risk for CROS as it places the condyles in a posterior position (18). Kerstens *et al.* pointed out the role of a posteriorly repositioned condyle in CROS as 87% of the condyles had a more posterior postoperative position (19).

This study aims to analyze the effectiveness of CBCT in the evaluation of the position, angulation and displacement of the condyles; evaluate the existence of differences in angulation, condylar position and intercondylar distance before and after orthognathic surgery; apply the results of this study to the clinical practice of orthodontists and maxillofacial surgeons.

The objective of this study was to analyze the effectiveness of CBCT in the evaluation of the condylar position, angulation and intercondylar distance before and after bimaxilary surgery. A further aim was to verify if bimaxillary surgery, using the Lefort I for maxillary impaction and counterclockwise rotation of the upper occlusal plane combined with the BSSO for mandibular advancement greater than 8mm, could cause significant changes in the mandibular condyles.

## Material and Methods

This retrospective study, approved by the Ethics Committee of the Faculty of Medicine of University of Coimbra (CE 62015 - according to the 1964 Helsinki declaration and is later amendments or comparable ethical standards), was performed using a sample of 20 adult patients, aged 27,0 ± 6,51 (18-41y), with a male/female ratio of 3/17, diagnosed with severe skeletal Class II and subjected to orthognathic surgery. The surgical techniques applied were Le Fort I osteotomy for impaction of the maxilla and conterclockwise rotation of the upper occlusal plane and BSSO for mandibular advancement. The magnitude of mandibular advancement was greater than 8 mm and less than 12mm (measured at B-point) and in all surgeries rigid internal fixation was performed using miniplates.

For all the individuals of the sample a CBCT was performed before (T1) and 8 weeks after surgery (T2), and the results were sent to observational and statistical study. The sample was selected based on the following inclusion criteria: individuals diagnosed with severe skeletal Class II, requiring orthognathic surgery, with surgical planning performed by the Department of Orthodontics – Faculty of Medicine, University of Coimbra- and with surgery performed at the Department of Maxillofacial Surgery (Coimbra Hospital and Universitary Centre). The exclusion criteria involve individuals with craniofacial syndromes (eg: clefts lip and palate); severe facial asymmetry; facial deformities secondary to trauma; degenerative joint disease; condylar fractures, condylar congenital anomalies or subjected to condilectomies; and individuals whose CBCT (pre, post-surgical or both) did not allow to evaluate the structures required in the study.

In this study, the pre and postoperative variables measured from the CBCT were the condylar angulation (measured on axial, frontal and sagittal plane); the intercondylar distance (measured in the axial plane); and the condylar position (measured in the sagittal plane).

All patients were positioned with the head in natural posture and the teeth in maximum intercuspation position with the tongue and lips in resting position. Volumetric data was analyzed with 3D-OS Nemoceph software (Software Nemotec SL, Madrid, Spain).

**Figure 1:**
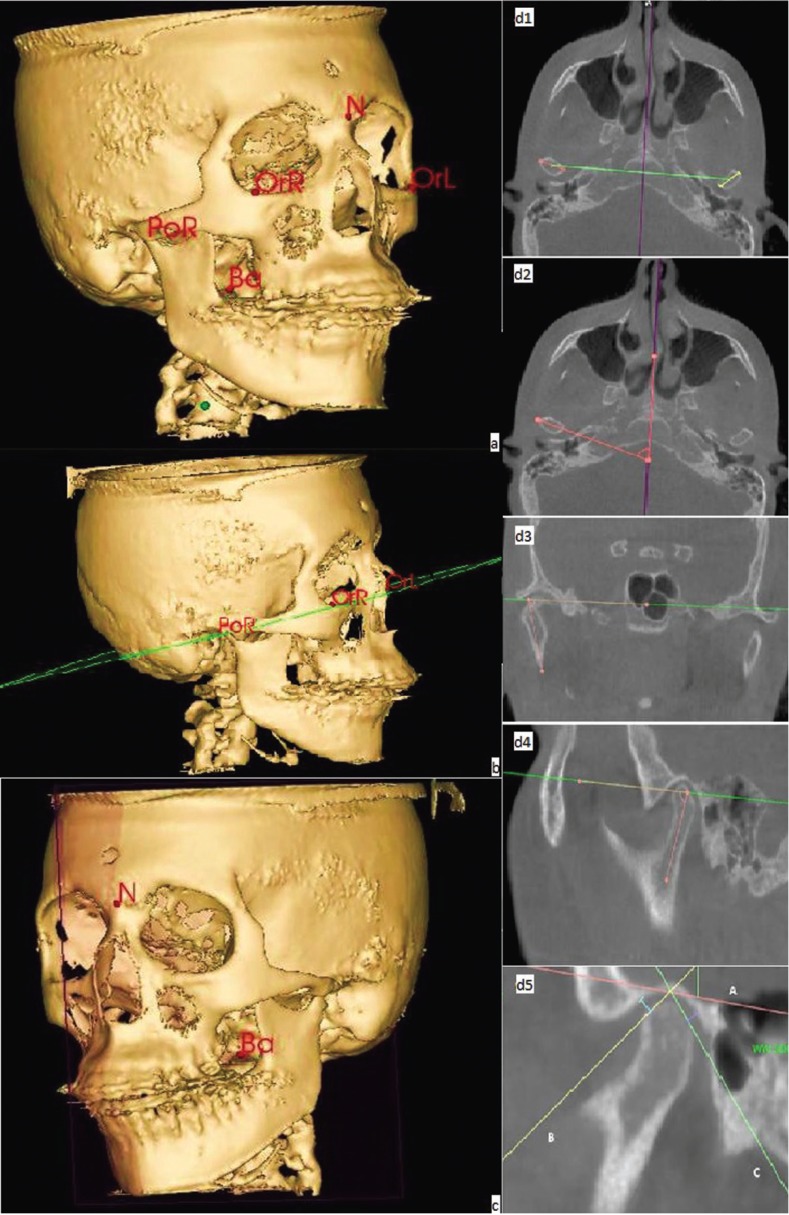
Midpoints of Reference (a); Frankfort Horizontal Plane (FHP) (b); Midsagittal Plane (MP) (c); Assessment of the intercondylar distance (d1), condylar angulation in the axial plane (d2), condylar angulation in the frontal plane (d3), condylar angulation in the sagittal plane (d4), condylar position in the sagittal plane (d5).

To perform the required measurements, first the midpoints of reference and plans for guidance were marked as shown in Figure 1. After marking the reference midpoints and defining the guiding plans (Fig. 1) the study variables were measured according to methods previously defined in the literature and served as a basis for this study (3,16,20).

The intercondylar distance was measured between the midpoint of the head of the right condyle and the midpoint of the head of the left condyle (Fig. 1).

The axial angle of the condyle was determined by the angle between the line connecting the medial and lateral pole of the condyle head and the midsagittal plane (Fig. 1). The frontal condylar angulation was determined by the angle formed by the axis of the vertical branch of the mandible and the FHP (Fig. 1). The sagittal condylar angle was determined by the angle formed by bisecting the anterior and posterior edge of the condyle head and the FHP (Fig. 1).

In the sagittal plane, the line A was drawn through the most upper surface of the glenoid fossa and parallel to the FHP. From this line, two tangents were drawn to the anterior and posterior borders of the condylar head (line B and C respectively). The measurement of anterior and posterior space was performed by a perpendicular line drawn from each previously marked tangent lines (Fig. 1). The position of the condyle in the glenoid fossa was determined by Pullinger & Hollender Formula as described in  Table 1 (17).

**Table 1:**
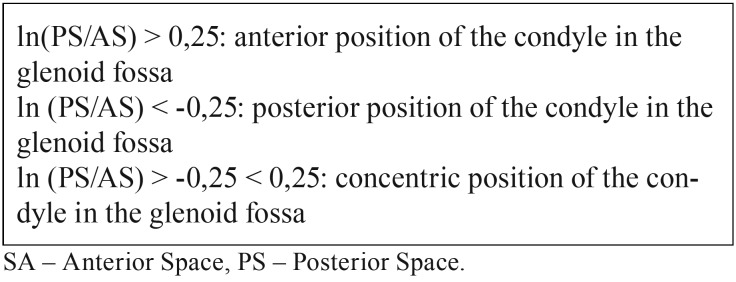
Condylar Position Classification by Pullinger & Hollender’s Formula.

All linear and angular measurements were performed by the same observer, assuming the rounding to the hundredths in the values obtained. In order to increase the accuracy of measurements and to reduce the bias of the sample, we used the method Kamelchuk *et al.* - each parameter was measured three times and only the average value of three measurements was considered for statistical purposes (18).

-Statistical Analysis

The statistical analysis was performed using the SPSS statistical platform (SPSS, Inc, Chicago, IL). To assess the possible existence of statistically significant differences, the use of paired tests was necessary, however, due to the large number of measurements, it increases the probability of error type I. In order to avoid this it was decided to conduct a MANOVA analysis designed to simultaneously measure all variables and then post-hoc tests using ANOVA. To assess if the position of the condyles remained the same before and after intervention Kappa statistics were calculated and the corresponding test. A chart was also compiled concerning the classification change.

This study adopted a significance level of 0.05 (α=0,05), corresponding to a level of confidence of 95%. The resulting distribution of values obtained for each of the variables is shown graphically, along with some statistics that are presented in the  Table 2.

**Table 2:**
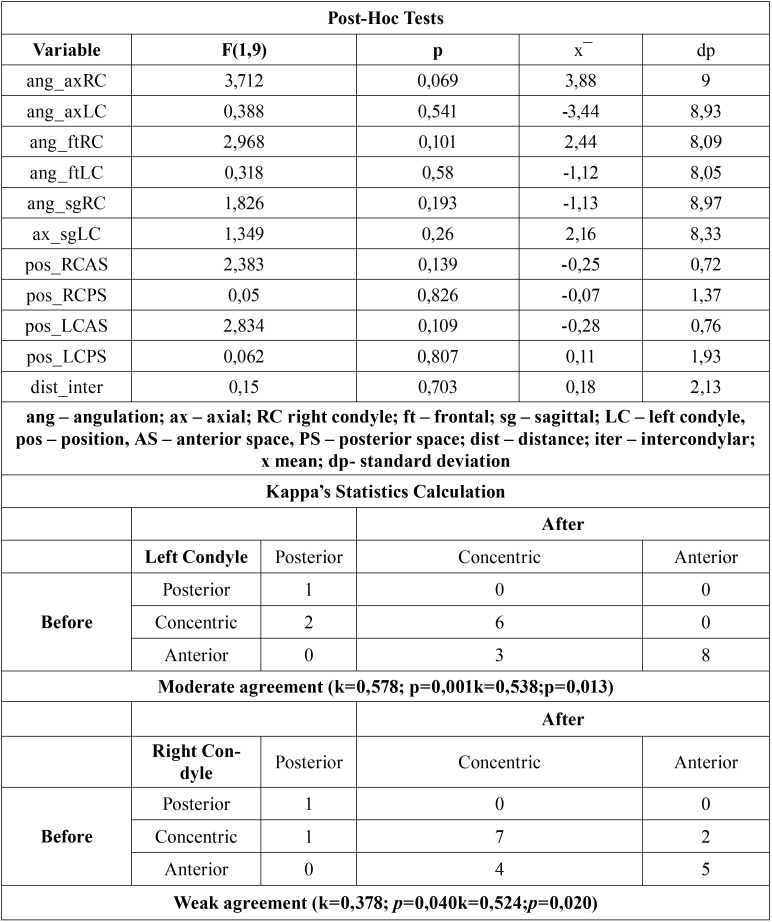
Results of post hoc tests.

## Results

In order to assess if there are statistically significant differences in the parameters under study, post hoc tests were performed for each of the variables. The results are presented in  Table 2. The level of significance assumed was 5 per cent.

There were no statistically significant differences (F_TP (1,10)=1,323;*p*=0,343) in any of the variables measured between T1 and T2. Note that, although there were slight variations in linear and angular measurements between the pre and post-surgical existing sample, these were extremely small, having no relevance from a statistical point of view.

**Figure 2:**
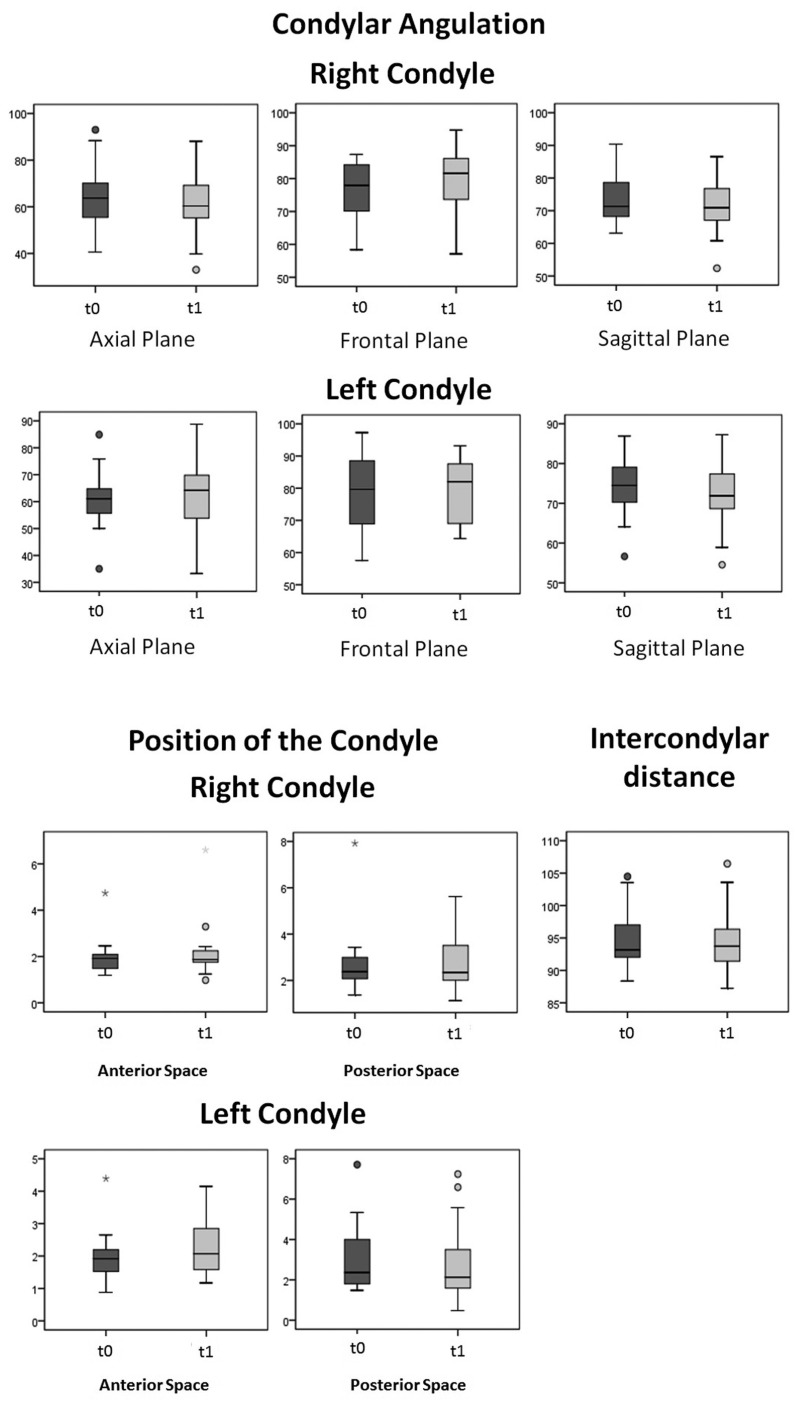
Diagram of extremes and quartiles referring to condylar angulation, position of the condyle (anterior and posterior joint space), and intercondylar distance.

The diagrams of extremes and quartiles referring to condylar angulation, position of the condyle (anterior and posterior joint space), and intercondylar distance, measured in T1 and T2, are presented in the Figure 2. At the axial level, the right condyle presented, on average, a slight reduction of its angulation (from 64,86º to 60,98º). The same didn’t occur to the left condyle, since their average angulation increased slightly in T2 (from 61,43º to 62,54º). There was then a lateral rotation of the right condyle, unlike the left condyle, which experienced a slight medial rotation. The angulation in the frontal plane showed, on average, an increased (from 76,14º to 79,58º the right condyle and from 78,43º to 79,56º the left condyle). Both condyles tended to execute a medial rotation movement. In the sagittal plane the average values of condylar angulation decreased. The right condyle went from 73,98º to 71,50º and the left condyle was reduced from 74,06º to 71,90º after the surgical phase. The condyles then suffered a posterior-inferior movement after surgery.

The intercondylar distance remained virtually constant, with an average range of 94.81 mm in T1 to 94.63 mm in T2.

Regarding the condyle position in the glenoid cavity, the anterior and posteior joint space showed changes in opposite directions. There was an increase of the anterior space in T2 (1.94 to 2.19 mm in the right condyle and 1.96 to 2.24 mm in the left condyle); the posterior space was maintained or reduced, going from 2.67 to 2.73 mm in the right condyle and from 2.95 to 2.84 mm in the left condyle. Applying the Pullinger & Hollender formula on the mean anterior and posterior space, it can be concluded that both condyles transited from a rating of “Anterior” at T1 to a rating of “Concentric” in T2 ( Table 3, Table 4) (17). This data allows us to predict that there is indeed a tendency to subsequent posterior movement of the condyles after surgery.

**Table 3:**
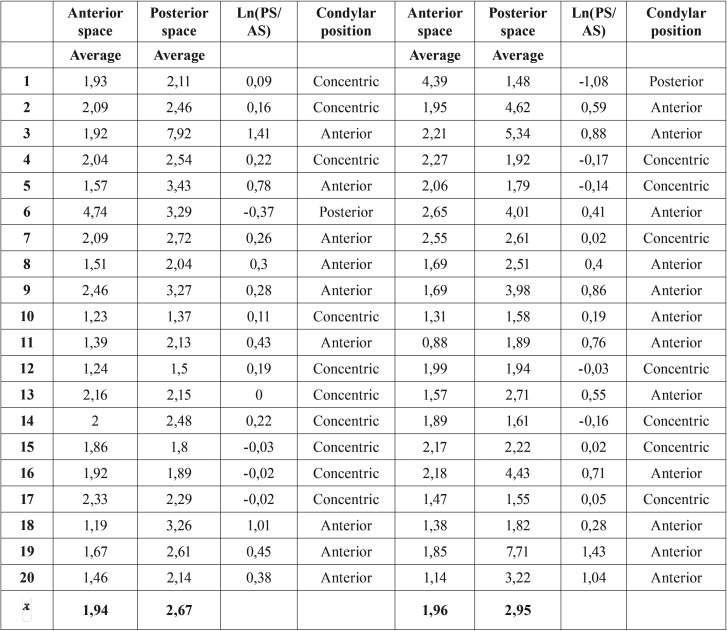
Measurement of the condyle position (Pre-surgical).

**Table 4:**
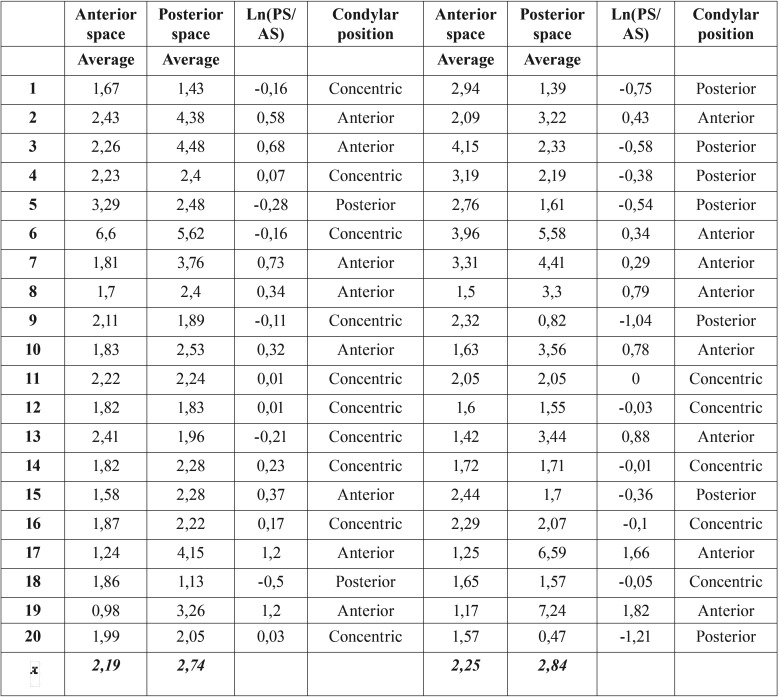
Measurement of the condyle position (Post-surgical).

**Figure 3:**
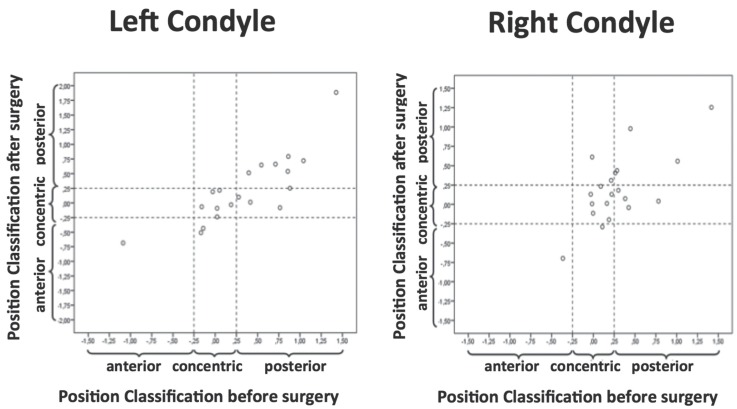
Dispersion diagram referring to the position classification of the left and right condyle before and after surgery.

Figure 3 shows the dispersion diagrams related to the classification of pre and postsurgical condylar position. The abscissa axis shows the classification of the position in T1 and the ordinate axis the classification obtained at T2. The classification uses as cut points -0.25 and 0.25, whereby the cases with values that lie within these limits is classified as concentric and outside these limits as anterior or posterior, as shown in the graph. The points that are located close to the diagonal represent cases where there was no change in the classification and the remaining points represent a change of classification.

Regarding the position of the condyles, it was verified that the left condyle shows moderate agreement (k=0,578; *p*=0,001) regarding the position classification at T1 and T2. Also the right condyle presents a weak agreement (k=0,378; *p*=0,040) regarding the position classification in T1 and T2. Combining this data with the post-hoc tests, it is possible to conclude that in most cases the condyles did not alter their position on the glenoid fossa.

## Discussion

Several authors state that the position of the condyles often suffers changes after orthognathic surgery (7,16). Among the factors responsible for topographic change of the mandibular ramus and condyles during surgery are: patients’ posture, the tension of the mastigatory muscles, the type of fracture, type of fixation used, the rigidity of the segments or the position of the condyle (11,16). Complications resulting from condylar displacement range from skeletal relapse to the disturbance of the occlusal stability causing temporomandibular disorders (7,15,16). Some authors suggest that rotational changes are closely related to progressive condylar resorption (14). Thus, it is consensual in the literature that progressive repositioning of the condyles ensures the stability of the surgical outcome, reduces the damaging effects on TMJ and can improve the postoperative mastication function (21,22). This has led to an increasing number of authors suggesting methods to avoid condylar displacement and radiographically monitorize the position of the condyles in patients undergoing orthognathic surgery (7,16,23).

In this study, the use of CBCT allowed the proper evaluation of the position and angulation of the condyles in axial, frontal and sagittal sections with high dimensional stability.

Regarding the condylar angulation, the data shows no statistically significant differences between the results obtained in T1 and T2. These results are in agreement with those obtained by Ueki *et al.* who claim that even when the condylar positioning device was not used, the angulation and position of the condyles changes little or nothing (24). Still, in the axial plane the angulation slightly reduced on the right condyle and increased on the left condyle, while in the frontal and sagittal planes both condyles respectively increased and decreased their average angulation.

Although frontal and sagittal condylar angulations have a greater tendency to remain constant, the same doesn’t seem to occur in the axial plane (4,7,9,12,16). Kim *et al.* concluded that the axial angles decreased with surgery, increasing further over time, although it was not statistically significant (12). In another study, the same authors noticed again the reduction of the axial angle, this time in patients submitted to mandibular sagittal split osteotomy (7). They justify the internal rotation of the head of the condyle to be due to a mandibular morphology in “V” and the location of the osteotomy lines.

Through the average angulation obtained in the axial plane, it’s possible to state that the right condyle tends to perform the internal rotational movement described previously. However, the same did not occur with the left condyle. Although there was no statistical significance, an extension of the sample in the future may help clarify the direction of rotation of the condyles on the axial plane.

Although a comparative analysis between single or bimaxillary surgery was not performed, there are studies in the literature that indicate that the condylar angulation in the axial and sagittal planes change significantly in patients undergoing orthognathic surgery in the maxilla and mandible (16). On the other hand, some authors mention that no significant differences exist between both surgical modalities, stating that the single-jaw procedure of the mandible can also be a very unstable intervention (14,25).

Regarding the intercondylar distance, the sample showed a slight reduction of the distance between the midpoint of each condyle. Still, the differences were not statistically significant. In a three dimensional analysis of skeletal Class III patients, Draenert *et al.* observed that there was no statistically significant changes in the intercondylar distance and intercondylar angles in patients undergoing mandibular setback surgery, with or without intervention in the maxilla (26). Kim *et al.* had the same conclusions (16). However, Kerstens *et al.* contradicted these results, stating that the intercondylar distance increases in the mandibular set forward surgery and decreases in the mandibular setback surgery (27). Lee and Park suggested that factors such as the fixation method and the surgical technique influence the intercondylar distance (9).

The sagittal plane is the most appropriate to evaluate the condyle-fossa relationship (4). In addition to the measurement of the anterior and posterior joint space, this study categorized the position of the condyle (in T1 and T2) as anterior, posterior or concentric, according to the Pullinger & Hollender formula (17). The differences between the anterior and posterior space pre and post-surgery were not statistically significant. In addition, according to the Kappa statistics there was a moderate agreement between the classification established in T1 and T2, on both condyles. By correlating this information, it was found that the sample maintained the condylar position after orthognathic surgery. Previous studies had similar findings (7). The Pullinger & Hollender formula proved thus to be a useful and viable method to classify the condylar position because the threshold values allow a sufficiently large window of results so that minimal changes in the condyle position don’t change the classification established in this study (17). However, when applying the same classification formula by sticking to the average values for the anterior and posterior joint space, both condyles pass from an anterior position in T1 to a concentric position in T2. Relating to the post-surgical reduction of the condylar angulation in the sagittal plane, it can be stated that the condyles tend to perform a posterior and inferior movement after orthognathic surgery.

This posterior-inferior movement is often referred in the literature (3,7,11,15). According to Chen *et al.* the posterior displacement of the condyles may be associated with the manual manipulation of the proximal segment during surgery (3). The adaptive properties of the soft tissues lead to changes in the position of the condyles, which tend subsequently to recover a more anterior and superior position. This recovery movement may be associated with the lengthening of the masticatory muscles and ligaments, the resolution of edema and the removal of the surgical splint (3,11,13).

Several long-term studies report the tendency of the condyles to return to the original position (3,8,11,13). Kim *et al.* indicate in a long-term study that the condyles move posteriorly up to 6 months after surgery and then recover their original position (7). Chen *et al.* showed slightly different results, indicating that the condyles recover their position through an anterior-superior movement, three months after surgery (3). In this study, the CBCT images were obtained on average 8 weeks after surgery, thus it’s only possible to characterize the displacement of the condyles soon after surgery. In order to assess the existence of a recovery movement of the condylar position and determine when this occurs, a CBCT control, over a long period of time, is necessary.

Cevidanes *et al.* demonstrated that the posterior displacement of the condyles in patients undergoing bimaxillary surgery is not significantly different from patients undergoing unimaxillar surgery (25). Furthermore, it has been proved that the condylar displacement is not statistically correlated to the advancement or mandibular setback (9). It must be noted that despite the tendency to move, the displacement of the condyles after orthognathic surgery is in most cases extremely small. In the literature it is accepted that when the condylar movements are below 2mm, the clinical effects are questionable (13).

However, the range of the condylar position displacement compatible with a postsurgical normal function is still not well-established (25). Proffit *et al.* have suggested that the changes that occur after surgery do not present a normal distribution and that considerable changes appear only in some patients (28). The orthognathic surgery changes the occlusion and the neuromuscular environment of the patients, requiring a period of adaptation (29). According to three-dimensional preliminary assessments of Carvalho *et al.* there is a marked individual variability in postoperative adaptation (30). Moreover, many patients can adapt to occlusion and condylar positions not considered ideal. The most superior and anterior condyle position along with a good, integrated muscle activity, with a great occlusal stability and the articular disc well interposed, has been considered the ideal state of the condyle. However, radiographically, the ideal depends on the thickness of the articular soft tissues, the tissue degeneration, and the mandibular posture and remodeling, since all these factors can affect the position of the condyle (7).

Future studies with larger sample sizes, long-term follow-ups and improved methodologies will probably be able to provide additional data regarding the position and angulation of the condyles and how it can affect bone remodeling and the adaptive capacity of the neuromuscular system.

## Conclusions

Despite the limitations inherent in this study, mainly due to the sample size and time of postoperative monitoring, some conclusions can be drawn:

There were no statistically significant differences in the measurements of the intercondylar distance, angulation and position of the condyles in patients undergoing orthognathic surgery, when comparing the pre- and post-surgical CBCT images.

 It was verified that the condyles move from a previous anterior position before surgery to a more concentric position after surgery, although there is no statistical significance when considering the average values of the angulation and condylar position. Combined with the reduction of the condyle head angulation in the sagittal plane, it is possible to conclude that the condyles tend to perform a posterior and inferior movement immediately after orthognathic surgery.

Finally, the present study showed a limited amount of condylar changes in patients submitted to large mandibular advancement (>8mm) with BSSO combined with Lefort I for maxillary impaction and counterclockwise rotation of the upper occlusal plane.
